# Genome-Wide Identification of Genes Involved in General Acid Stress and Fluoride Toxicity in *Saccharomyces cerevisiae*

**DOI:** 10.3389/fmicb.2020.01410

**Published:** 2020-06-25

**Authors:** Nichole R. Johnston, Sunitha Nallur, Patricia B. Gordon, Kathryn D. Smith, Scott A. Strobel

**Affiliations:** ^1^Department of Molecular Biophysics and Biochemistry, Yale University, New Haven, CT, United States; ^2^Department of Chemistry, Yale University, New Haven, CT, United States

**Keywords:** fluoride, acid, genes, yeast, vesicle-transport, vacuole, nutrient uptake

## Abstract

Hydrofluoric acid elicits cell cycle arrest through a mechanism that has long been presumed to be linked with the high affinity of fluoride to metals. However, we have recently found that the acid stress from fluoride exposure is sufficient to elicit many of the hallmark phenotypes of fluoride toxicity. Here we report the systematic screening of genes involved in fluoride resistance and general acid resistance using a genome deletion library in *Saccharomyces cerevisiae*. We compare these to a variety of acids – 2,4-dinitrophenol, FCCP, hydrochloric acid, and sulfuric acid – none of which has a high metal affinity. Pathways involved in endocytosis, vesicle trafficking, pH maintenance, and vacuolar function are of particular importance to fluoride tolerance. The majority of genes conferring resistance to fluoride stress also enhanced resistance to general acid toxicity. Genes whose expression regulate Golgi-mediated vesicle transport were specific to fluoride resistance, and may be linked with fluoride-metal interactions. These results support the notion that acidity is an important and underappreciated principle underlying the mechanisms of fluoride toxicity.

## Introduction

Responsiveness to acid stress is an essential adaptation for microorganisms. Unlike multicellular organisms in which internal, sensitive tissues are protected from toxicants, microbes are directly exposed to their environment. As a result, a microbe’s survival is dependent on the evolution of proteins and pathways capable of mitigating any potential stressors. Fungi have proven quite successful in combating acid stress in particular; yeast grow optimally in pH 4.0–6.0 media, and have been reported to efficiently adapt to media with a pH as low as 2.5 (Narendranath and Power, 2005; Liu et al., 2015).

Eukaryotic microbes, such as fungi, contain organelles for the compartmentalization and specialized functions in combating acid stress. The intracellular pH of the cytoplasm and organelles are tightly controlled by H^+^-ATPases, particularly Pma1p, and V-ATPases (Kane, 2016). This regulation is critical for maintaining the function of intracellular proteins, many of which are sensitive to pH changes. Of particular sensitivity are transmembrane and metabolic enzymes, such as phosphate transporters, and phosphofructokinase (Hardewig et al., 1991; Ding et al., 2013). During acidosis, fungi lower metabolism and protein synthesis, and increase the production of saturated lipids and ergosterol to adjust membrane fluidity (Sousa et al., 2012; Brandao et al., 2014; Guo and Olsson, 2016; Godinho et al., 2018; Palma et al., 2018). Cells also undergo remodeling of their plasma membranes and cytoskeletal components (Mollapour et al., 2006; Guo et al., 2018). While these are general response mechanisms to acidosis, different acids often elicit unique stress phenotypes based on their given properties.

Fungi routinely encounter acids in natural environments. Strongly acidic environments feature charged ions that cannot readily cross biological membranes without protein transporters. Consequently, exposure of fungi to highly acidic extracellular environments disrupts the electrochemical potential and function of the fungal plasma membrane (Carmelo et al., 1996; Russell and Gould, 2003; Johnston and Strobel, 2019). In contrast, exposure of fungi to weak acids can result in intracellular acidification (Mira et al., 2010b). Weak acids, such as carbonylcyanide p-trifluoromethoxyphenylhydrazone (FCCP), and 2,4-dinitrophenol (DNP), act as protonophores, shuttling protons across biological membranes (Loomis and Lipmann, 1948; Benz and McLaughlin, 1983; Kenwood et al., 2014; Geisler, 2019). Prolonged exposure to FCCP and 2,4-DNP uncouples oxidative phosphorylation of the mitochondria (Loomis and Lipmann, 1948; Brennan et al., 2006). While this was presumed to be due to proton shuttling across the mitochondrial membrane and the consequent disruption of mitochondrial membrane potential, it was recently found that the phenotype was dependent on the acidified cytoplasm (Berezhnov et al., 2016). Given that different acids cause distinct toxicity phenotypes, studying the cellular response to an acid gives insight into its mechanism of toxicity.

Hydrofluoric acid is a weak acid with a pK_a_ of 3.2 and is highly abundant in the environment. Fluoride inhibits metabolism, acidifies the cytoplasm, and elicits oxidative stress in cells (Feig et al., 1971; Kawase and Suzuki, 1989; Hamilton, 1990; Barbier et al., 2010). The mechanism behind these phenotypes has been assumed to be linked with the high affinity of fluoride for metals. *In vitro*, fluoride has been demonstrated to sequester the metals from the active site of essential metalloproteins, but only at millimolar fluoride concentration (Adamek et al., 2005). Our lab recently discovered that *Saccharomyces cerevisiae* lacking fluoride exporters undergoes cell cycle arrest at 50 μM NaF, well below the concentration required for any known metalloprotein inhibition (Li et al., 2013). We consequently found that fluoride induced cytoplasmic acidosis in yeast, resulting in a disruption of membrane potential and nutrient uptake (Johnston and Strobel, 2019). From this, the question becomes: What aspects of fluoride toxicity are the direct consequence of acid stress?

Yeast genomic libraries serve as powerful tools for high-throughput screens. Genetic knockout libraries, encapsulating the deletion of each nonessential gene in the yeast genome, can be used to identify genes essential for a particular function (Giaever and Nislow, 2014). In the context of acid tolerance, deletion of any gene involved in crucial resistance pathways will result in sensitivity to that acid. By comparing the genetic resistance pathways for acids of known toxicity with those for fluoride, we can identify both overlapping essential genes, and genes that are specific to fluoride.

Here we report the analysis of genes important for fluoride resistance, and compare them to genes involved in reducing extracellular and intracellular acidosis. We selected acids that do not chelate strongly to metals to distinguish acid stress effects from inhibition of metalloproteins. We report that several genes linked to Golgi-vesicle transport are unique to fluoride toxicity. Nonetheless, there is significant overlap in essential genes between fluoride and general acid stress.

## Materials and Methods

### Media, Strains, and Knockout Library

The yeast strain used in this study was BY4741. Yeast were typically grown in YPD buffer, containing 1 *g* yeast peptone (Becton, Dickinson and Co., Franklin Lakes, NJ, United States), 0.5 *g* yeast extract (Becton, Dickinson and Co.), 50 μL of 1% adenine (Sigma), and 2.5 mL of 40% glucose (Sigma) per 50 mL total volume in water. YPD-agar plates consist of YPD, plus an additional 1 gr agar per 50 mL solution (Becton, Dickinson and Co.). Sodium fluoride (Sigma Aldrich), HCl (Sigma Aldrich, St. Louis, MO, United States), H_2_SO_4_ (Sigma Aldrich), 2,4-DNP (Sigma Aldrich), and FCCP (Sigma Aldrich) were used in the reported experiments. The knockout library is commercially available (Dharmacon, Lafayette, Colo, United States). Knockout strains were inoculated overnight in YPD, then incubated on YPD agar +/- acid at starting O.D. 0.67 at 30°C until fully grown, typically 48 h. Some genetic deletions resulted in slower growth, and required addition time.

### Liquid Growth Assay and Serial Dilutions

Liquid growth assays were conducted over 24-h intervals, as described in Li et al., 2013. Agar plates used in serial dilutions were placed in the 30°C incubator for 48 h before imaging. Serial dilutions were prepared using the standard protocol.

### Determining pH_intra_ and pH_extra_

Cells were grown in 2 mL YPD ± acid at starting O.D. 0.1. After 4 h of growth at 30°C, the cells were harvested by spinning and resuspending in PBS. The pH of the YPD buffer (pH_extra_) was measured using a pH probe. The pH of the cells (pH_intra_) was measured using 5(6)-carboxyfluorescein diacetate (CFDA) dye under its standard protocol. The pH was determined by comparing fluorescence of each cell to a standard curve of yeast permeabilized using 70% ethanol, then resuspended in PBS with pH ranging from 3.5-7.5.

### Bioinformatics Analysis of Genetic Knockout Screen

Genes were grouped by pathway based on their descriptions on the *Saccharomyces* Genome Database (yeastgenome.org). Network clusters were generated using the String Enrichment app (RRID:SCR_005223), MCode (RRID:SCR_015828), and ClueGO (RRID:SCR_005748) on Cytoscape (version 3.7.2). Enrichment ratios and *p*-values were generated using WebGestalt (http://www.webgestalt.org, RRID:SCR_006786). Values were transferred to Prism Software for graphing (RRID:SCR_002798).

### Monitoring ROS Production

Yeast (wild type or ΔVMA11) were grown to log phase, and then added at O.D. 0.5 to 1.5 mL YPD +/- acids. The acids were at the IC_50_ concentration: 75 mM NaF, 20 μM FCCP, 0.3 mM 2,4-DNP, 120 mM HCl, and 30 mM H_2_SO_4_. Cells were shaken at 30°C for 6 h, and then spun and washed three times in PBS. Yeast were resuspended in 100 μL PBS and 10 μM dihydroethidium dye, shaken, and incubated in the dark for 5 min. Relative fluorescence units were measured using a plate reader, *ℷ*_excitation_ = 480 nm and *ℷ*_emmission_ = 580 nm. Fold change was calculated by comparing fluorescence of each sample to the average fluorescence of wild type yeast grown in YPD for 6 h, adjusted by cell count.

### Assessing Glucose Uptake

Yeast were grown for 4 h at 30°C starting at O.D. 0.5 in 2 mL YPD +/- acids. The acids were tested at the IC_50_ for each: 75 mM NaF, 20 μM FCCP, 0.3 mM 2,4-DNP, 120 mM HCl, and 30 mM H_2_SO_4_. Cells were then washed three times in PBS. Yeast were resuspended in 100 μL PBS with 1 μL 2-NBDG, a fluorescent glucose mimic. Yeast were placed into a water bath at 30°C for 1 h, washed three times in PBS, and resuspended into 100 μL PBS. Fluorescence was analyzed with a plate reader at *ℷ*_excitation_ = 494 nm and *ℷ*_emmission_ = 521 nm, and adjusted by cell count.

### Monitoring Intracellular Polyphosphate With ^31^P NMR

*In vivo* NMR was conducted with yeast grown to log phase, as outlined in Johnston and Strobel, 2019.

## Results

### Non-essential Genetic Deletion Screen for Enhanced Acid Sensitivity

We set out to distinguish which aspects of fluoride toxicity are most likely associated with acid stress rather than metalloprotein inhibition. In order to establish this distinction, we compared the results from fluoride exposure to those for exposure to HCl, H_2_SO_4_, FCCP, or 2,4-DNP; each of which elicit acid stress, but do not have a high affinity for metals.

**FIGURE 1:**
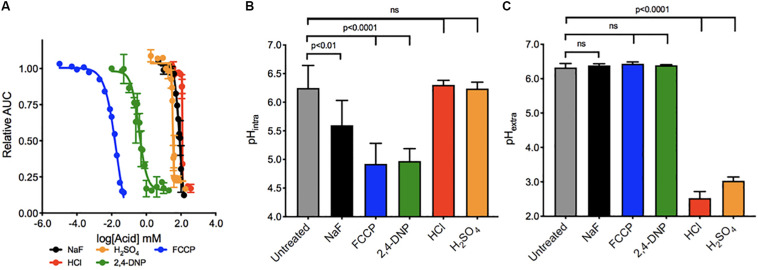
Toxicity of acids in yeast. **(A)** The toxic range of each acid in inhibiting yeast growth is assessed over 24 h by a liquid growth assay, with increasing concentrations of each acid. Yeast were then exposed to acids at their IC_50_ concentrations for 4 h, and the **(B)** intracellular and **(C)** extracellular pH of the yeast was established using pH-sensitive dye and a pH electrode, respectively. *P* values are denoted above, and were calculated using Prism software.

We first determined the concentration range of each acid required to elicit growth arrest over 24 h in wildtype yeast (Figure 1A). The IC_50_’s ranged broadly between the acids, with the most potent being FCCP, and 2,4-DNP (0.02 and 0.3 mM, respectively) and the least being sulfuric acid (30 mM), sodium fluoride (75 mM), and hydrochloric acid (120 mM). The addition of weak acids FCCP, 2,4-DNP, and NaF causes intracellular acidification, but not extracellular acidification (Figures 1B,C). These acids have positive pK_a_s of 6.2, 4.1, and 3.2, respectively. Consequently, a subset of each acid would be in its protonated form inside the cell and able to pass through lipid compartments. In contrast, the strong acids HCl and H_2_SO_4_ with pK_a_s of -6.3 and -2.0, respectively, do not exist in their protonated form, and only cause extracellular acidification. In this way, fluoride is the most like 2,4-DNP and FCCP. It does not significantly alter the pH of the media, and induces intracellular acidification at 4 h exposure. However, the degree of intracellular acidification for fluoride is not as great as 2,4-DNP or FCCP at their respective IC_50_’s.

A commercially available *S. cerevisiae* knockout library was used to identify genes that confer resistance to acid stress. This library consists of single deletions in 5,250 non-essential genes. We exposed all 5,250 yeast knockout strains to the lowest observed adverse effect level (LOAEL) and IC_25_ concentrations of each acid on YPD agar plates and allowed the strains to grow to saturation. Across all five acids, a total of 4,908 genetic deletions caused no noticeable sensitivity to stress. 342 genetic deletions resulted in significant growth arrest under acid exposure (Supplementary Table 1). 133 of genetic deletions resulted in sensitivity to fluoride, 132 genetic deletions resulted in sensitivity to H_2_SO_4_, while 161 total deletions resulted in sensitivity to HCl. Exposure to either of the weak acids FCCP or 2,4-DNP was sensitized by 204 or 148 genetic deletions, respectively.

**FIGURE 2:**
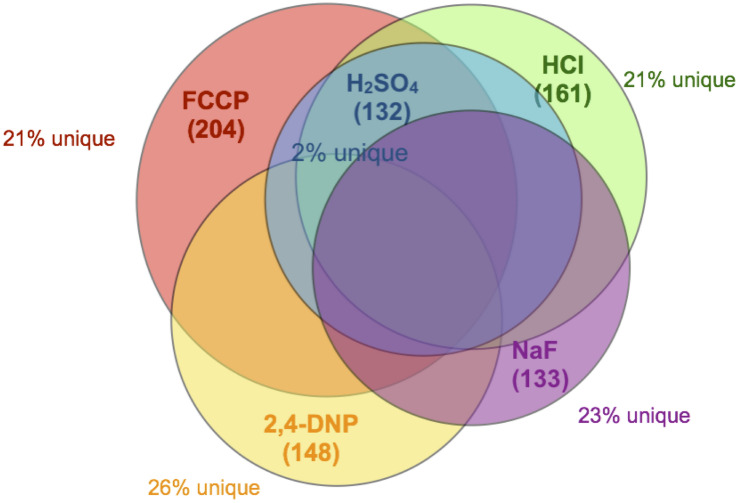
Venn diagram of overlapping genes. Each circle encompasses the full set of gene deletions resulting in sensitivity to NaF (133 genes, purple), FCCP (204 genes, red), 2,4-DNP (148 genes, yellow), HCl (161 genes, green), or H_2_SO_4_ (132 genes, blue). Also noted in the figure is the percent of those genes that do not overlap with any other dataset.

The overwhelming majority of genes caused sensitivity to more than one acid. Of the 132 genes conferring resistance to sulfuric acid, less than 2% were specific to sulfuric acid (Figure 2). The other acids had roughly 20% unique genes related to toxicity resistance, including fluoride. In other words, approximately 80% of genes conferring resistance to fluoride are involved in resistance against other acids that lack metal affinity.

**FIGURE 3:**
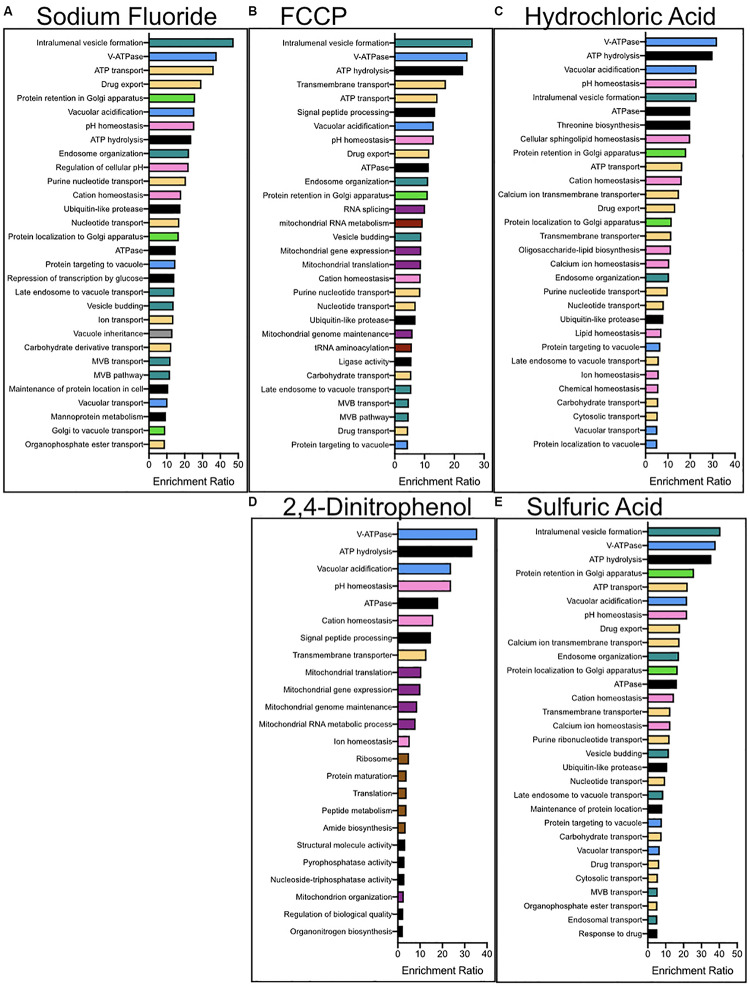
Enrichment analysis for the biological processes and molecular functions of genes conferring resistance to **(A)** NaF, **(B)** FCCP, **(C)** 2,4-DNP, **(D)** HCl, and **(E)** H_2_SO_4_ stress. Enrichment analysis was performed using WebGestalt, and redundant categories were eliminated. The top 30 most enriched pathways are reported above, having at least an enrichment ration value of 2 and a *p*-value below 0.01. The colors corresponding to the columns are (green) – golgi, (pink) – ion homeostasis, (purple) – mitochondria, (red) – nucleus, (yellow) – protein transporters, (brown) – ribosomes, (blue) – vacuole, (turquoise) – vesicle-mediated transport, and (black) – other.

We performed enrichment analysis using a serious of programs in order to determine the biological pathways, organelles, and genes most important for acid resistance. First, we utilized the functional analysis tool WebGestalt to calculate the enrichment of biological processes and molecular functions within each dataset (Figure 3). Datasets of sensitized genetic deletions for each of the five acids were enriched for those involved in vacuolar ATPase, pH maintenance, and vesicle formation. Genes involved in fluoride resistance were the most enriched for Golgi function. Genes involved in resistance to FCCP and 2,4-DNP were heavily enriched for mitochondrial function and DNA maintenance, as well as protein translation for 2,4-DNP resistance. Genes involved in resistance to strong acids HCl and H_2_SO_4_ were heavily enriched in ion homeostasis and cell surface genes, including nutrient transporters.

We then used the String Database to plot all 342 genes based on their corresponding protein-protein interaction networks (Supplementary Figure 1). Genes with a high degree of interaction were those associated with the ribosome and vacuole. Genes involved in fluoride tolerance were heavily enriched in vacuolar and Golgi interaction networks. Conversely, genes involved in weak acid tolerance were enriched in vacuolar and ribosomal networks, and those in strong acid tolerance were enriched in vacuolar and cell surface networks.

**FIGURE 4:**
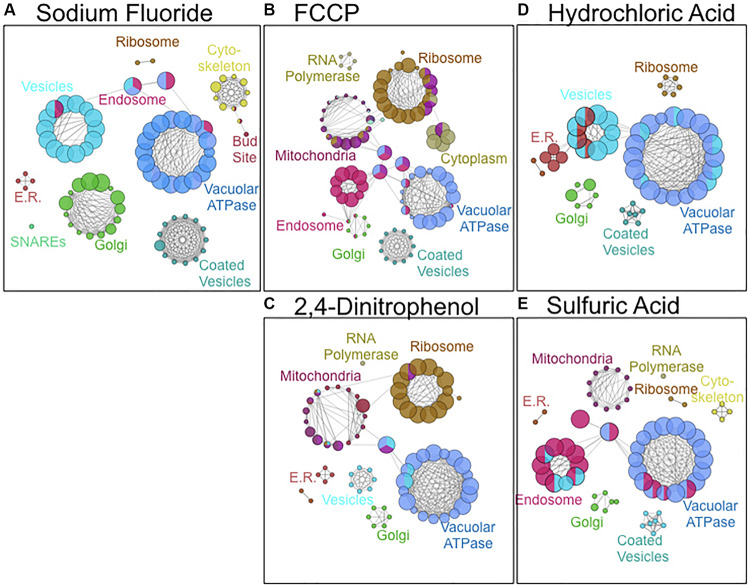
Cellular components involved in resistance to **(A)** NaF, **(B)** FCCP, **(C)** 2,4-DNP, **(D)** HCl, and **(E)** H_2_SO_4_ stress. Images were composed using ClueGO software from pathways with *pV* ≤ 0.050. Node size is proportional to the fraction of genes in that particular node.

As a third method of bioanalysis, we utilized ClueGO to examine important cellular components of acid resistance (Figure 4). Genes involved in mitochondrial and ribosomal function were enriched in response to weak acid stress caused by 2,4-DNP and FCCP exposure. Genes involved in endosomal and vesicle-mediated transport processes were enriched in response to the strong acids HCl and H_2_SO_4_. The vacuolar ATPase genes – essential in pH maintenance – resulted in high sensitivity in response to exposure to any of the acids. Fluoride toxicity was selectively enhanced upon deletion of genes involved in cytoskeleton, vesicle-mediated transport, and Golgi function. Genes involved in Golgi, coated vesicles, and SNARE function were more enriched in response to fluoride stress than any other acid.

Of the 342 gene deletions that affected acid sensitivity, 39 deletions caused sensitivity to all six acids tested. Within these genes were significant enrichment for vacuolar ATPase and pH maintenance, vesicle-mediated transport, and glycoprotein production (Supplementary Figure 2A). 101 of 342 gene deletions affected both weak acids 2,4-DNP and FCCP, and 106 gene deletions affected both strong acids HCl and H_2_SO_4_. Of the 133 genetic deletions resulting in sensitivity to fluoride, 32 of those genes – ARF1, CCC2, COX20, CRZ1, ERG24, GOS1, HOF1, IES6, KES1, MNN10, NHX1, NPR1, PEX17, PRS5, ROX3, RPP1A, RVS167, SAC1, SLT2, SRN2, STP22, VAM3, VAM7, VPS8, VPS9, VPS24, VPS28, VPS36, VPS61, VRP1, YDR455C, and YOR041C – were unique to fluoride resistance. Gene ontology analysis of these genes showed significant enrichment for Golgi function, ESCRT machinery, and SNARE receptor activity (Supplementary Figure 2B).

Fluoride resistance is heavily influenced by the expression of two fluoride transporters, FEX1, and FEX2. Our lab previously found that single deletions of fluoride transporters do not affect fluoride tolerance (Li et al., 2013). As expected, the single deletions did not appear in our screen to confer sensitivity to any acid. Conversely, deletion of both genes resulted in over 1000-fold increased sensitivity to fluoride. There is a possibility that Golgi function is necessary to successfully incorporate fluoride transporters to the cell membrane. To test this, we inserted plasmids containing GFP-FEX1 into 14 of the genetic deletions specific for fluoride resistance (Supplementary Figure 3). Microscopy analysis of the yeast demonstrated strong localization of the GFP signal to the plasma membrane, indicating the successful incorporation of FEX1. Furthermore, the genetic deletions specific to fluoride resulted in less than 10-fold increased fluoride sensitivity, as opposed to the 1000-fold observed by completely abolishing FEX expression. This is not necessarily unexpected, as genetic deletions that would completely abolish transmembrane protein incorporation would mostly likely be lethal. These data suggest that the genes involved in Golgi function cause sensitivity unrelated to FEX localization.

### V-ATPase Confers Acid Resistance Through pH and ROS Maintenance

**FIGURE 5:**
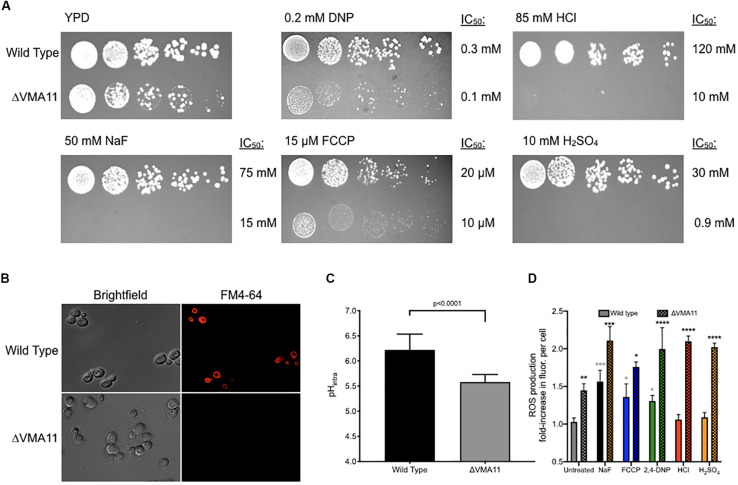
Sensitivity of ΔVMA11 mutation in yeast. **(A)** Serial dilutions of wild type and ΔVMA11 yeast on agar plates containing normal YPD, and YPD with NaF, HCl, FCCP, CH_3_COOH, and H_2_SO_4_. The IC_50_’s of each were calculated using the 24-h liquid growth assay (data not shown). **(B)** Imaging of vacuolar membrane using FM4-64 dye. Cells are grown to log phase. **(C)** Intracellular pH of wild type and ΔVMA11 yeast after 4 h of growth, as assessed using 5(6)-CFDA. *P*-value is shown; *p*-value was calculated using GraphPad Prism Software. **(D)** Change in reactive oxygenated species production in yeast grown 6 h, in YPD or YPD with acids. Astericks represent statistical significance, as calculated using prism software. Gray asterisks represent the comparison between untreated and acid treated wild type cells, while black asterisks represent the statistical significance between acid treated wild type, and acid treated ΔVMA11 yeast cells.

Deletion of V-ATPase subunits resulted in significant sensitivity to all of the acids tested. This suggests that vacuolar ATPase is essential for general acid resistance. V-ATPase is a proton pump composed of 13 subunits. Deletion of 12 of the 13 subunits caused significant sensitivity to acid stress, and deletion of 10 of those subunits resulted in sensitivity to all six acids. Of these, deletion of VMA11 resulted in the greatest acid sensitivity (Figure 5A). ΔVMA11 yeast had a 5-fold lower IC_50_ to fluoride than wild type, two-fold to FCCP and 2,4-DNP, and ten-fold and 35-fold lower to HCl and H_2_SO_4_, respectively. We tested for V-ATPase function in ΔVMA11 yeast by monitoring the vacuolar electrochemical potential (Figure 5B). V-ATPase is essential for maintaining the pH gradient between the cytosol and vacuole; consequently, disruption of V-ATPase disrupts this gradient (Huynh and Grinstein, 2007; Maxson and Grinstein, 2014). We monitored vacuolar electrochemical potential using the dye FM4-64, which enters the cells via vesicle trafficking and incorporates into the vacuolar membrane after 1 h of exposure in normal cells. In ΔVMA11 yeast, we found no evidence of vacuolar staining, indicating a loss of V-ATPase activity.

Several labs have reported that inhibition of V-ATPase results in cytoplasmic acidosis (Martínez-Muñoz and Kane, 2008; Diab and Kane, 2013). We monitored intracellular pH at 4 h of growth in ΔVMA11 yeast, and found that the pH dropped from 6.2 to 5.6 (Figure 5C). pH maintenance is an essential mechanism for acid resistance. However, disruption in intracellular pH maintenance might predictably make cells more sensitive to molecules that further disrupt intracellular pH. Conversely, ΔVMA11 yeast were the most sensitive to strong acids HCl and H_2_SO_4_, which primarily alter extracellular pH. This result suggests that V-ATPase might function beyond intracellular pH maintenance in resisting acid toxicity.

Several reports have linked vacuolar function with oxidative stress resistance, although the mechanism is still unknown (Milgrom et al., 2007; Diab and Kane, 2013; Charoenbhakdi et al., 2016; Nishikawa et al., 2016). Deletion of V-ATPase subunits in yeast results in hypersensitivity to a wide variety of oxidative stresses. We monitored levels of reactive oxygenated species in yeast, both under acid stress, and in V-ATPase knockout, and found that deletion of VMA11 was sufficient to increase intracellular reactive oxygenated species independent of acid stress (Figure 5D). Addition of acids resulted in increased ROS production in wild type yeast, and even greater addition in ΔVMA11 yeast. HCl and H_2_SO_4_ did not significantly induce ROS production in wild type, but did in ΔVMA11 yeast. While it is already well established that V-ATPase functions in pH maintenance, its role in lowering cytoplasmic ROS may also contribute to its function in general acid resistance.

### Vesicle-Mediated Endocytosis Affects Nutrient Uptake During Acid Stress

Our lab previously found that fluoride-activated acidosis disrupts the electrochemical gradient of the plasma membrane and initiates nutrient starvation signaling (Johnston and Strobel, 2019). While yeast upregulate the expression of nutrient scavengers under fluoride stress, the transmembrane nutrient transporters are inhibited by the disrupted pH gradient. This leads to the question of how the yeast are able to uptake nutrients with inhibited protein transporters.

After vacuolar ATPase function, the next highest enriched processes involved in general acid resistance in this screen were vesicle formation, trafficking, and endocytosis. Vesicle trafficking and endocytosis are induced during acid stress (Ben-Dov and Korenstein, 2013). Endocytosis is involved in both transmembrane protein recycling and nutrient uptake from the extracellular environment (Grant and Donaldson, 2009; Antonescu et al., 2014; Hinze and Boucrot, 2018). Consequently, disruption of essential genes involved in endocytosis resulted in cell sensitivity to nutrient depletion, particularly glucose, and amino acids (Jones et al., 2012; Lang et al., 2014). Given that acid exposure decreases transmembrane nutrient transporter activity in yeast, we hypothesized that yeast utilize endocytosis during acid-induced nutrient starvation for the uptake of nutrients.

**FIGURE 6:**
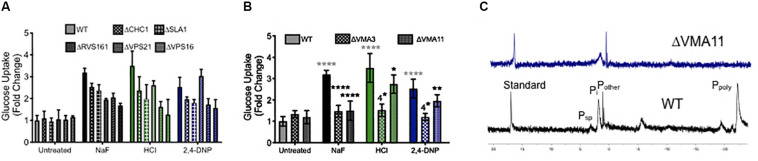
Role of endocytosis, vesicle trafficking and V-ATPase in nutrient uptake. Glucose uptake (monitored using the fluorescent dye 2-NBDG) after 4 h exposure to acids in wild type (WT) and **(A)** endocytosis and vesicle trafficking knockouts and **(B)** V-ATPase subunit knockouts. **(C)** ^31^P NMR of wild type and ΔVMA11 yeast. Statistical significance analysis for **(A)** had *p* < 0.0001 for every value of acid treatment compared to wild type, or acid treatment of wild type compared with genetic knockouts. The only exception for this was ΔRVS161 yeast, which had *p* < 0.001 difference in sugar uptake compared with wild type. Statistical analysis for **(B)** is represented in the graph, whereby gray asterisks represent the comparison between untreated and acid treated wild type cells, while black asterisks represent the statistical significance between acid treated wild type, and acid treated V-ATPase knockout yeast cells.

To test this hypothesis, we monitored glucose uptake during acid stress using 2-deoxy-2-[(7-nitro-2,1,3-benzoxadiazol-4-yl) amino]-D-glucose (2-NBDG), a fluorescent glucose mimic (Figure 6A). As expected, exposure to fluoride induced enhanced 2-NBDG uptake, as did exposure to both strong and weak acids. Deletion of genes involved in endocytosis did not alter 2-NBDG uptake under normal conditions. However, those yeast knockout strains showed reduced uptake of 2-NBDG in the presence of fluoride or other acid, despite being more sensitized to the acid stress. The yeast strain lacking VPS16– a gene involved in the tethering and fusion of endosomes – had the most significant reduction in 2-NBDG uptake under all conditions. Together, these data support the hypothesis that endocytosis is involved in nutrient uptake during acid stress.

We also tested 2-NBDG uptake in V-ATPase genetic knockouts, and saw a similar reduction in intracellular 2-NBDG (Figure 6B). This can be either due to reduced sugar uptake, or a reduction in its storage. V-ATPase is essential for regulating the pH gradient necessary for vesicle fusion and endocytosis (Geyer et al., 2002; Lafourcade et al., 2008; Brett et al., 2011; Maxson and Grinstein, 2014). Beyond that, V-ATPase is essential for vacuolar activity, including the storage of ions. One particularly important stored ion is phosphate, which is used by the vacuole to sequester metals (Nguyen et al., 2019). High concentrations of stored phosphate have been demonstrated to resist oxidative stress in yeast (Lev et al., 2017; Johnston and Strobel, 2019). We assessed phosphate storage using ^31^P NMR, and as expected, no vacuolar polyphosphate could be detected in V-ATPase mutants, and the overall concentration of intracellular phosphate was lower than wild type (Figure 6C). In all, V-ATPase appears to function in acid resistance through oxidative stress resistance, pH maintenance, vesicle trafficking, and nutrient storage.

## Discussion

Research into the mechanics of fluoride toxicity has seen increased interest with the concern of public safety in fluoridated water. Fluoride is naturally abundant throughout our ecosystem, and additionally supplied in government-regulated water at 0.7–1.2 ppm (40–60 μM) to increase our bone health. However, too much fluoride results in toxicity. While the exact cause of intracellular fluoride toxicity is unknown, the downstream stress phenotypes are well established. Fluoride elicits oxidative stress, metabolic inhibition, and intracellular acidosis (Barbier et al., 2010; Agalakova and Gusev, 2011). This has been long presumed to be due to metalloprotein interactions with fluoride, particularly glycolytic enzymes such as enolase (Marquis, 1995). However, HF is also an acid, which has underappreciated biological consequences. Here we report the screening of 5,250 non-essential *S. cerevisiae* genes for their involvement in acid resistance using a deletion library. The results demonstrate a heavy enrichment of genes involved in vacuolar function and vesicle-mediated transport for general acid resistance. Genes involved in resistance to fluoride (tested at 35 and 50 mM) overlapped largely with those of other acids, but had a higher enrichment in genes involved in Golgi function than the other acids.

Out of the 5,250 genes tested in this knockout screen, 32 gene deletions resulted in sensitivity to only fluoride. Of these, three genes (VPS61, YOR041C, and YDR455C), are putative open reading frames with unknown function. However, all three overlap in the genome with genes that could confer fluoride resistance. VPS61, whose deletion causes vacuolar defects, overlaps with RGP1, part of the Golgi membrane exchange factor. YOR041C overlaps with CUE5, coding a ubiquitin-binding protein involved in autophagy signaling. YDR455C overlaps with the Na^+^/H^+^ exchanger NHX, which itself conferred resistance to fluoride toxicity and is required for osmotolerance. Other genes involved uniquely in fluoride tolerance were the copper transporter CCC2, the cytochrome c oxidase gene COX20, stress transcription factor CRZ1, and the DNA repair gene IES6. Each of these were not part of a conserved pathway of genes related to fluoride resistance, but most probably function in general stress resistance. Fluoride is well established to cause metal imbalance, DNA damage, oxidative stress, and metabolic arrest; expression of these genes would counteract these effects (He and Chen, 2006; Fina et al., 2014; Johnston and Strobel, 2019).

The most significantly enriched pathway unique to fluoride resistance was Golgi function and vesicle-mediated transport. Among these were genes involved in endocytosis and cell surface maintenance, including ERG24, HOF1, RVS167, VPS9, and VRP1. Also unique to fluoride were subunits of the endocytosis tethering complexes ESCRT and CORVET. Endocytosis was found to confer resistance against all acids tested in this report. Why these five genes caused sensitivity only to fluoride, is not immediately clear. Fluoride has previously been reported to selectively inhibit vesicle trafficking, both through metal- and G-protein-interactions (Matsuo et al., 1998; Stow and Heimann, 1998; Taraschi et al., 2001; Barbier et al., 2010). As such, it could be that these sets of genes are particularly sensitized to fluoride as opposed to other acids. Supporting the hypothesis that vesicle-transport is most sensitized to fluoride exposure, many genes essential for SNARE and Golgi function conferred resistance to only fluoride. Metallo-fluoride also reversibly disrupts Golgi stacking and inhibits essential Golgi GTPases (Finazzi et al., 1994; Back et al., 2004). One particular known target, Arf1p GTPase, was among the genetic deletions that caused sensitivity only to fluoride toxicity (Lanoix et al., 2001). Given that these genes are unique in affecting fluoride resistance and there is scientific precedent that metallo-fluoride alters their activity, Golgi and vesicular trafficking are likely specific targets of fluoride.

Genes involved in general protein turnover, including peroxisome function, amino acid synthesis, and ribosomal function, are involved in fluoride resistance. Stress, in general, is rescued by functional protein turnover, which can degrade inhibited or counterproductive proteins and synthesize proteins that combat stress. However, fluoride is known to inhibit ribosomes (Ravel et al., 1966; Vesco and Colombo, 1970; Hardesty et al., 1973). Fluoride also elicits oxidative stress, which independently halts translation (Shenton et al., 2006). Given that acid stress is rescued by protein turnover, non-essential genes that aid in protein synthesis could provide significant tolerance to fluoride.

The majority of genes that conferred significant resistance to fluoride toxicity also conferred resistance to acids lacking high metal affinity. These genes were largely enriched for involvement in V-ATPase and vesicle-mediated transport. Both of these have a multitude of functions that could potentially aid in acid resistance. For instance, V-ATPase functions in pH maintenance, endocytosis regulation, and nutrient storage (Maxson and Grinstein, 2014). We also demonstrate that inhibition of V-ATPase resulted in an increase of intracellular ROS independent of additional stressors. As many acids – including fluoride – cause oxidative stress, loss of ROS maintenance would predictably enhance toxicity. Endocytosis is also involved in many processes, including cell surface turnover and protein recycling (Goode et al., 2015). Endocytosis has been previously reported to confer resistance to acid stress, and conversely, both alkaline and acid stress partially inhibit endocytosis (Sandvig et al., 1987; Pereira et al., 2012; Ben-Dov and Korenstein, 2013). We report here that endocytosis is involved in the uptake of nutrients during pH disruption. Given that many acids facilitate pH disruption along the plasma membrane, one might predict that endocytosis is involved in general acid resistance.

Other laboratories have reported using genetic knockout libraries to investigate acid stress. In this study, we compared fluoride with acids whose stress is primarily attributed to pH imbalance. We also compared the genes involved in fluoride resistance with previously published genetic screens for sensitivity to formic, propionic, acetic, and sorbic acids (Mollapour et al., 2004; Mira et al., 2009, 2010a; Henriques et al., 2017). These acids cause a broader range of toxicity phenotypes compared to strong acids or protonophores. Phenotypes include oxidative stress, nutrient starvation, and metabolic inhibition (Kitanovic et al., 2012; Liu et al., 2012; Stratford et al., 2020). Consequently, the published gene lists have significantly less overlap between these wide-range acid toxicants compared with more similar acids, such as HCl and H_2_SO_4_ (Supplementary Figure 4). Acetic acid was the most unique, with 400–500 more gene deletions conferring significant growth defects compared to other acids. Importantly, fluoride was the most similar to other acids, with only 25% unique genes. Of the 75% shared genes, the majority were shared by either acetic acid or sorbic acid. This is consistent with our observation that roughly 80% of genes conferring resistance to fluoride also function in rescuing from general acid stress. Formic, propionic, acetic, and sorbic acids each have a much higher proportion of unique genes compared with fluoride, which suggests that they have more specific mechanisms of toxicity.

Due to the limitations of a genetic knockout library, we are not able to assess the involvement of essential genes in general acid resistance. This includes the glycolytic protein enolase, which has long been presumed to be a key target of fluoride toxicity, because it is an essential gene and not available in the screen. However, we are able to investigate the role of nonessential genes involved in metabolism to identify the overlap between fluoride and general acid toxicity resistance. These nonessential genes, whose deletion would lower but not inhibit metabolism, did not significantly impact fluoride, nor general acid resistance. This suggests that the ability of a cell to metabolize at an optimal rate does not influence acid tolerance. In support of this hypothesis, the Nislow lab conducted a loss-of-function screen of 87.1% essential yeast genes for fluoride sensitivity, and found only 13 that conferred significant sensitivity (Yan et al., 2008). Of these, half were involved in lipid biosynthesis and vesicle-mediated transport, and none were involved in carbohydrate metabolism.

Fluoride toxicity has long been attributed to metal interactions *in vivo.* While metal binding is undoubtedly a factor, the role of acid stress has been underappreciated. Here, we report that the majority of nonessential genes involved in fluoride resistance are also involved in general acid resistance, particularly pH maintenance and vesicle transport. Acid stress is commonly encountered in the wild. As such, it would confer a significant evolutionary advantage for organisms to retain a widespread resistance mechanism. Overall, these data suggest that a significant factor in fluoride toxicity can be attributed to its properties as an acid.

## Data Availability Statement

All datasets generated for this study are included in the article/Supplementary Material.

## Author Contributions

NJ, KS, and SS contributed conception and design of the study. NJ, SN, and PG performed experimental work and statistical analysis. NJ and SS wrote the manuscript. All authors contributed to manuscript revision, as well as read and approved the submitted version.

## Conflict of Interest

The authors declare that the research was conducted in the absence of any commercial or financial relationships that could be construed as a potential conflict of interest.
