# Supplementary material for: Genome-Wide Identification of Genes Involved in General Acid Stress and Fluoride Toxicity in Saccharomyces cerevisiae
Source: Front Microbiol. 2020 Jun 25;11:1410. doi: 10.3389/fmicb.2020.01410 (PMC7329995; doi:10.3389/fmicb.2020.01410)
Supplement: Supplementary file 1 [file Data_Sheet_1.PDF]

Supplementary Material

1 Supplementary Figures

1.1 Supplementary Figure 1: Protein-protein interaction networks of genes whose deletion confer sensitivity to acid.

Supplementary Figure 1: Protein-protein interaction networks of genes whose deletion confer sensitivity to acid. Networks were plotted on Cytoscape using the String Database, and colored by their classification using the Yeast Genome Database Gene Ontology Slim Term Mapper. All 342 genes are plotted and colored in (A), and in later figures all color is removed except for genes that confer resistance to (B) NaF, (C) FCCP, (D) 2,4-DNP, (E) HCl, and (F) H_2SO_4.

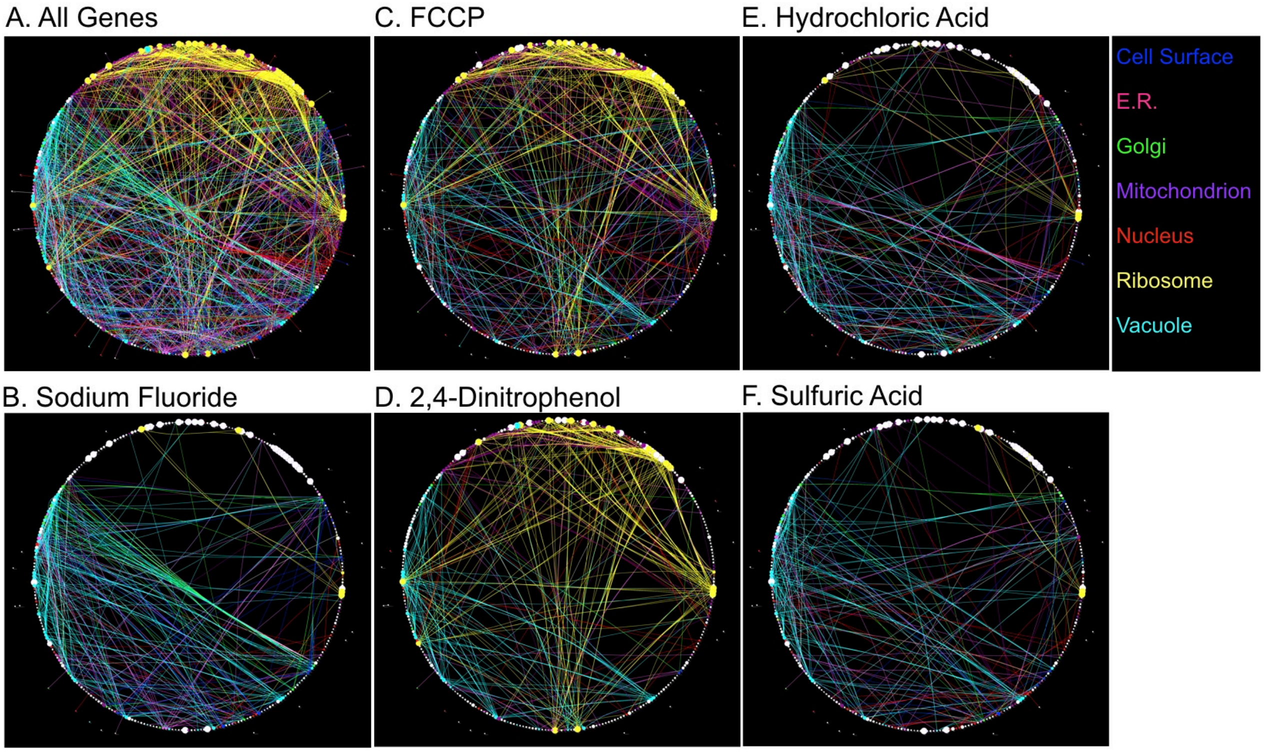

1.2 Supplementary Figure 2: Cellular components involved in resistance to

Supplementary Figure 2: Cellular components involved in resistance to (A) all acids, and (B) only NaF. Images were composed using ClueGO software from pathways with pV ≤ 0.050. Node size is proportional to the fraction of genes in that particular node.

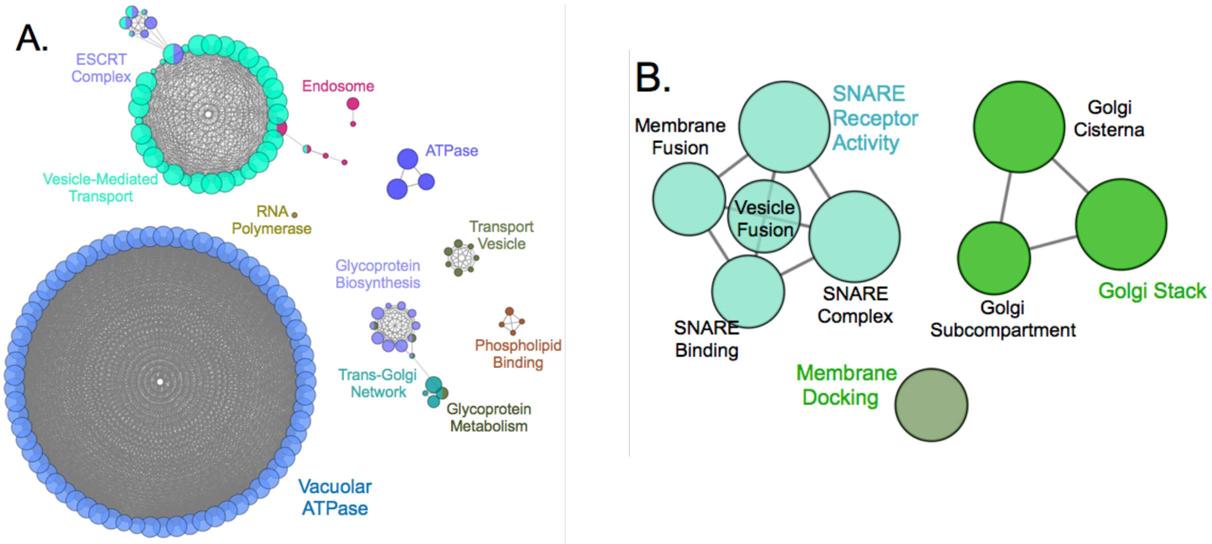

1.3 Supplementary Figure 3: Images of GFP-tagged FEX inserted into the denoted genetic knockout.

Supplementary Figure 3: Images of GFP-tagged FEX inserted into the denoted genetic knockout. 14 genetic knockouts that were transformed with a plasmid containing GFP-tagged FEX1. All cells showed localization of the GFP to the plasma membrane, suggesting that FEX1 has been successfully incorporated.

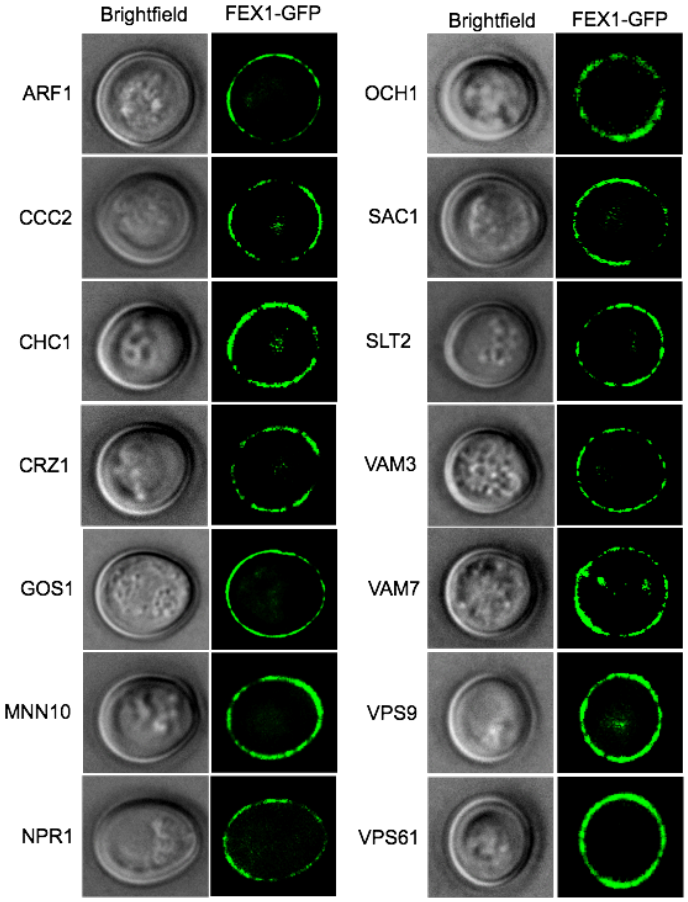

1.4 Supplementary Figure 4: Venn diagram of genes conferring sensitivity to fluoride or other acids.

Gene lists are compiled from Mollapour *et al.* 2004, Mira *et al.* 2009, Mira *et al.* 2010, and Henriques *et al.* 2017. Below is the same data represented as either a (A) area-proportional, (B) Edward's, or (C) classical Venn diagram.

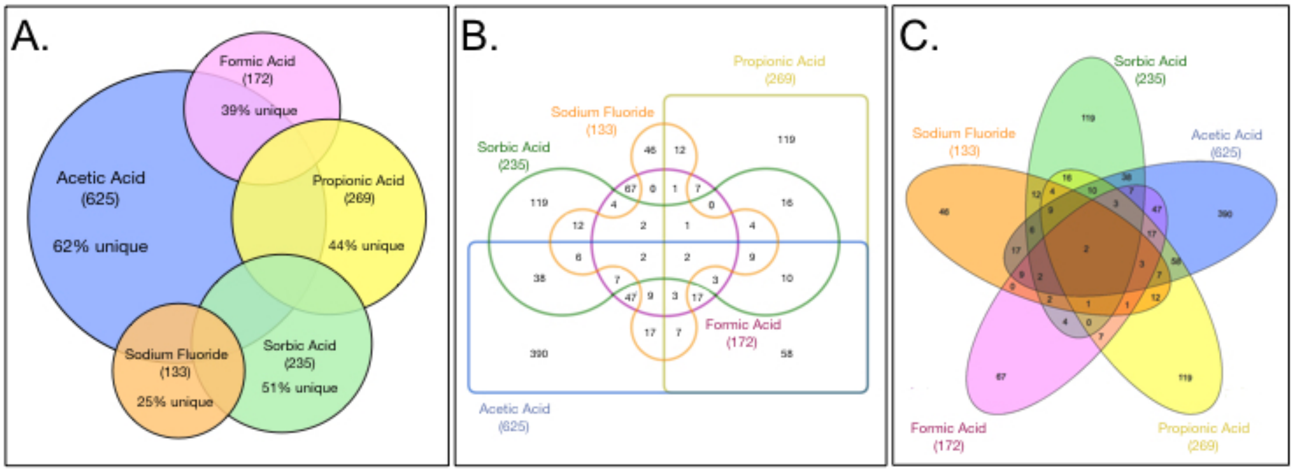

2 Supplementary Tables

2.1 Supplementary Table 1:

Gene deletions that confer significant sensitivity to acid exposure. Genes are grouped alphabetical under their corresponding cellular processes, and cellular processes are listed from most to fewest genes. Lower case "x" denotes sensitivity to acid only at the higher acid concentration, while upper case "X" denotes sensitivity at both the high and low acid concentrations.

Name	ORF name	FCCP	DNP	H_2SO_4	HCl	NaF
**<u>1. Mitochondria</u>**						
AEP1	YMR064W		x			
AFG3	YER017C	X	X	X	X	x
ATP7	YKL016C	X		x		
ATP17	YDR377W	x				
ATP22	YDR350C	X				
ATP25	YMR098C		x			
CBP2	YHL038C	X			X	
CCM1	YGR150C	X				
CEM1	YER061C	X				
COQ6	YGR255C	X	X			
COQ8	YGL119W	X	X			
COQ10	YOL008W	X				
COX10	YPL172C	X		X	X	
COX11	YPL132W	X				
COX18	YGR062C	X		x		
COX20	YDR231C					X

COX23	YHR116W	X		X		
CYC3	YAL039C	X		X	X	x
CYT2	YKL087C	x		x		
GEM1	YAL048C	X	x	X	X	x
GEP4	YHR100C	X	X			
GGC1	YDL198C	X	X		x	x
IBA57	YJR122W	X				
IMG1	YCR046C	X	x			
IMG2	YCR071C	x				
IMP2	YMR035W	X		X		
ISA2	YPR067W	x	x			
LIP2	YLR239C	x				
MBA1	YBR185C				x	
MDJ1	YFL016C		x			
MEF1	YLR069C	X	X			
MRF1	YGL143C		X			
MRP1	YDR347W	X	X		x	
MRP7	YNL005C	X	X			
MRP17	YKL003C	X				
MRP20	YDR405W	X				
MRP21	YBL090W	X	x	X	X	x
MRP51	YPL118W	X			X	
MRPL4	YLR439W	x	x			
MRPL6	YHR147C	x				
MRPL7	YDR237W	X				x
MRPL8	YJL063C	X				
MRPL9	YGR220C	X				
MRPL13	YKR006C		x			
MRPL15	YLR312W-A	x				
MRPL16	YBL038W	X	x	X	X	x
MRPL17	YNL252C	X	x			
MRPL23	YOR150W		x			
MRPL27	YBR282W	x				
MRPL32	YCR003W	X	x			
MRPL36	YBR122C		x			
MRPL37	YBR268W		x			
MRPL40	YPL173W	X	x	X	X	
MRPS5	YBR251W	X	X			
MRPS35	YGR165W	x				
MRX14	YDR115W	X	X			

MSD1	YPL104W	X			X	
MSF1	YPR047W	X				
MSM1	YGR171C	X				
MSR1	YHR091C	X	x	x		
MSS116	YDR194C	x				
MSW1	YDR268W		x			
MSY1	YPL097W	X	x			
MTF1	YMR228W	X	x			x
MTG1	YMR097C		x			
NAM2	YLR382C	X	x			
OXA1	YER154W	X	x			
PCP1	YGR101W	X	x	X		
PET54	YGR222W	X	X			
PET100	YDR079W	X	x	X		
PET117	YER058W	X				
PET123	YOR158W		x			
QRI5	YLR204W	X		x		
QRI7	YDL104C	X	x			
RIP1	YEL024W			x	x	
RML2	YEL050C	X	X			
RPO41	YFL036W	X				
RSM7	YJR113C	X	X			
RSM18	YER050C	X				
RSM19	YNR037C	x	x			
RSM22	YKL155C		x		x	
RSM24	YDR175C	x				
RSM27	YGR215W	X	X	X		x
SLM5	YCR024C				x	
SLS1	YLR139C	X	x			
SSQ1	YLR369W		X			
SOV1	YMR066W		x			
TIM11	YDR322C-A		x			
YTA12	YMR089C	X	X			

2. Vesicle-Mediated Transport

BRO1	YPL084W	X		X	x	X
CDC50	YCR094W	x		X	x	X
CHC1	YGL206C	X	X	X	X	X
CLC1	YGR167W	X	X	X	X	X
DID4	YKL002W	X		x	x	X
DRS2	YAL026C				x	

ERV14	YGL054C		x			
KES1	YPL145C					x
PEP3	YLR148W			X	X	X
PEP12	YOR036W	X	X	X	X	X
RVS167	YDR388W					x
SHE4	YOR035C	x		X	x	X
SNF7	YLR025W	X		X		X
SNF8	YPL002C	X		X	X	X
SRN2	YLR119W					X
STP22	YCL008C					X
SWF1	YDR126W			x	X	x
VAM3	YOR106W					X
VPS3	YDR495C			X	X	X
VPS4	YPR173C	X		X		X
VPS5	YOR069W			x	x	
VPS8	YAL002W					X
VPS9	YML097C					X
VPS16	YPL045W	x		X	X	X
VPS20	YMR077C	x		x	X	X
VPS21	YOR089C	X				X
VPS24	YKL041W					X
VPS25	YJR102C	X		X	x	X
VPS27	YNR006W	X		X	x	X
VPS28	YPL065W					X
VPS33	YLR396C		x			
VPS36	YLR417W					X

<u>3. Metabolism/Nutrient Transport</u>

ARO1	YDR127W		x	x	x	
ARO2	YGL148W			x	x	
CSF1	YLR087C	x		X	X	
DUG2	YBR281C				x	
GLY1	YEL046C		x			
HAP2	YGL237C	x	X			
HAP3	YBL021C			X	X	
HAP5	YOR358W	X				
HOM6	YJR139C				x	
ILV1	YER086W				x	
KRE6	YPR159W				x	
PFK1	YGR240C	X	x		x	
PRS3	YHL011C	X		X	X	x

PRS5	YOL061W					x
REG1	YDR028C				x	
RIB1	YBL033C		x			
THR1	YHR025W				x	
THR4	YCR053W				x	
TPS1	YBR126C			x		
TRP3	YKL211C		x			
YGP1	YNL160W	x		X	x	x
ZWF1	YNL241C			X	X	X

4. Ion homeostasis

AGP2	YBR132C	X	X	X	x	X
AQR1	YNL065W	X		X	x	
ARN1	YHL040C	X				
CCC2	YDR270W					x
CCH1	YGR217W	x		x	X	
DAL5	YJR152W	x				x
ECM7	YLR443W				x	
GUP1	YGL084C	x		X	x	
JEN1	YKL217W	x				
MAC1	YMR021C	X	X			x
MCH5	YOR306C	x			X	X
MID1	YNL291C			X	X	
NHX1	YDR456W					X
NPR1	YNL183C					x
PDR12	YPL058C	X				x
PDR18	YNR070W			X	X	x
SKY1	YMR216C	x		x		X
TPO2	YGR138C	X		x		
TPO3	YPR156C	X				
TRK1	YJL129C				x	

5. Vacuole

PKR1	YMR123W	X	x	x	x	x
RRG1	YDR065W		X			
VAM7	YGL212W					X
VMA1	YDL185W		x		x	X
VMA2	YBR127C	x	x	X	x	X
VMA3	YEL027W	X	x	X	x	X
VMA5	YKL080W	x	X	x	X	X
VMA6	YLR447C	X	x	X	X	X

VMA7	YGR020C	x	X	x	x	X
VMA8	YEL051W		X	x	x	X
VMA9	YCL005W-A	x	X	x	x	X
VMA10	YHR039C-B	X	X	X	x	X
VMA11	YPL234C	X	X	X	X	X
VMA13	YPR036W	x	x	X	x	x
VMA16	YHR026W	x	X	X	x	X
VMA21	YGR105W	x	X	x	x	X
VMA22	YHR060W	X	X	x	x	X
VPH2	YKL119C	X	X	x	x	X
VPS1	YKR001C	X		x	X	X
VPS15	YBR097W			x	X	X

6. Protein expression (Translation)

ALG3	YBL082C				x	
ALG6	YOR002W				x	
ALG8	YOR067C				x	
BUD23	YCR047C			x	x	
CAX4	YGR036C	x	x	x	X	X
DCS1	YLR270W	X	x	X		
DHH1	YDL160C				x	
GIR2	YDR152W				x	
MEF2	YJL102W	X	x			
NCL1	YBL024W	X	x			X
OST3	YOR085W				x	
POP2	YNR052C	X	x	X	X	X
REF2	YDR195W	x	x		x	
RPL12B	YDR418W		x			
RPP1A	YDL081C				x	x
SAC3	YDR159W	x		x	x	
SED1	YDR077W			x	x	
SLM3	YDL033C	X	X	x	x	
TEF4	YKL081W		x			X

7. Stress response

ATG4	YNL223W			X	X	X
BCK1	YJL095W			x	X	
BOL2	YGL220W	X	x	x	x	
CNB1	YKL190W				x	
CRZ1	YNL027W					x
DBF2	YGR092W				x	

GON7	YJL184W	X		X	X	X
GPH1	YPR160W	X				
HOG1	YLR113W	x		X	X	
HRK1	YOR267C	x				
KCS1	YDR017C	x	x	x	x	X
KSS1	YGR040W	X				
PBS2	YJL128C				x	
PDE2	YOR360C	X		X	x	
RVS161	YCR009C		X			X
SNG1	YGR197C		x			
WAR1	YML076C	X				

<u>8. DNA regulation</u>

FYV6	YNL133C	x		X	X	x
FZO1	YBR179C	X	X			
GEP5	YLR091W	X	x			
HTA1	YDR225W	x			X	
HTB2	YBL002W		x			
HTL1	YCR020W-B	X	x	X	X	x
IES2	YNL215W	X		x	x	X
IES6	YEL044W					X
LCL3	YGL085W	X				
MEC3	YLR288C	X	X	X		
MGM101	YJR144W	x				
MHR1	YDR296W		x			
MIP1	YOR330C	X				
MSH1	YHR120W	X	X	X	X	x
RAD6	YGL058W	X				
SHU2	YDR078C	X	x	X		

<u>9. Gene Expression (Translation)</u>

AIM10	YER087W	X	X			
BUR2	YLR226W			x	x	
CCR4	YAL021C			X	X	X
CDC40	YDR364C	x	x	X	x	X
CYC8	YBR112C	x	X	X	x	x
HFI1	YPL254W	X	X	x		
MOT2	YER068W	x		X	x	X
ROX3	YBL093C					X
RPB4	YJL140W	x				
RPH1	YER169W		x			

TAF14	YPL129W	X	X	X	x	X
TFB5	YDR079C-A	x	X	X		
TPD3	YAL016W	x	x	x	x	X
URE2	YNL229C	X				

10. Golgi

ARF1	YDL192W					X
ANP1	YEL036C	X	X	X		X
COG1	YGL223C	X	x	X	X	X
GLO3	YER122C	x				
GOS1	YHL031C					x
GYP1	YOR070C		x			
HUR1	YGL168W	X		X	x	x
MNN10	YDR245W					x
OCH1	YGL038C	x	x	X	X	X
PMR1	YGL167C	X		X	x	X
SAC1	YKL212W					x
VPS52	YDR484W	x	x		X	X
VPS53	YJL029C	X		x	x	X
VPS54	YDR027C	X		x	x	X

11. Lipid metabolism

ARE2	YNR019W	X				
CHO1	YER026C	X	X	X	x	X
ELO2	YCR034W	x				
ERG2	YMR202W	x				X
ERG3	YLR056W		x		x	
ERG4	YGL012W		X			X
ERG24	YNL280C					X
LCB5	YLR260W	X		X		
LEM3	YNL323W		X			
MCT1	YOR221C	x				
TSC3	YBR058C-A	X	X	X	X	
VPS34	YLR240W			X	X	X

12. Growth

BEM1	YBR200W	X	x	X	X	X
BUD25	YER014C-A	x		x	x	X
ILM1	YJR118C				x	
IME4	YGL192W				x	
PET130	YJL023C	x				

RHO4	YKR055W	X	x	X	x	X
RSR1	YGR152C	x	x			X
SIN3	YOL004W	x		x	x	X
SIT4	YDL047W			x	x	
**13. Endoplasmic reticulum**						
CSG2	YBR036C			x	x	
DIE2	YGR227W				x	
EUG1	YDR518W	X	x			
GET1	YGL020C	X	X		x	
GET2	YER083C	x	x	x	x	x
PER1	YCR044C				x	
SBH1	YER087C-B		x			
**14. Protein regulation**						
ADD37	YMR184W	X	x			
APE2	YKL157W	X				
DAP2	YHR028C		x			
DOA4	YDR069C	X		X	x	X
DOC1	YGL240W		x			
KEX2	YNL238W	X	X		x	
PEX17	YNL214W					X
**15. Cell surface**						
DCW1	YKL046C				x	
GAS1	YMR307W	X		X	X	
PMP3	YDR276C	x		X	x	X
SCW10	YMR305C			X	x	
SLT2	YHR030C					X
SMI1	YGR229C				x	
**16. Cytoskeleton**						
CNM67	YNL225C		x			
HOF1	YMR032W					X
NIP100	YPL174C	X			x	
SLA1	YBL007C	X		X	x	x
VRP1	YLR337C					X
**17. Unknown**						
BRP1	YGL007W				x	
FAP1	YNL023C	x	x	x	x	x

FYV8	YGR196C				x	
RRG7	YOR305W		x	x	x	
RRG9	YNL213C	X	x	x	X	
SKG3	YLR187W				x	
VPS61	YDR136C					X
VPS63	YLR261C	x			x	
VPS69	YPR087W	X		x	x	x
YBL044W	YBL044W	X	x			
YBL094C	YBL094C				x	
YCL001W-B	YCL001W-B		x			
YDR008C	YDR008C		x			
YDR114C	YDR114C	X	x			
YDR169C-A	YDR169C-A		x			
YDR182W-A	YDR182W-A		x			
YDR455C	YDR455C					X
YGL007C-A	YGL007C-A		x	x	x	X
YGR035W-A	YGR035W-A	x	x	X	x	X
YGR219W	YGR219W		x			
YHL015W-A	YHL015W-A		x			
YKL096C-B	YKL096C-B	x	x		x	
YKL118W	YKL118W	x	X	x	x	X
YKL158W	YKL158W	X		X		
YMR320W	YMR320W				x	
YNL170W	YNL170W		x			
YNL184C	YNL184C				x	
YOR041C	YOR041C					X
YOR331C	YOR331C		x	x		X
YPL119C-A	YPL119C-A	X	X			
YPR099C	YPR099C	x		X	X	X
YPR172W	YPR172W				x	
